# A National Cancer Database Study on the Demographic, Prognostic, and Socioeconomic Factors Affecting Survival in Adenocarcinoma in Villous Adenoma

**DOI:** 10.7759/cureus.82367

**Published:** 2025-04-16

**Authors:** Beau Hsia, Yanick Tade, Susan Rafie, Gejla Toromani, Peter T Silberstein, Deepak Vadehra, Timothy J Brown

**Affiliations:** 1 Medicine, Creighton University School of Medicine, Phoenix, USA; 2 Surgery, Creighton University School of Medicine, Omaha, USA; 3 Osteopathic Medicine, Midwestern University Arizona College of Osteopathic Medicine, Glendale, USA; 4 Biology, Miami University, Oxford, USA; 5 Oncology, Creighton University School of Medicine, Omaha, USA; 6 Medical Oncology, Roswell Park Comprehensive Cancer Center, Buffalo, USA; 7 Abramson Cancer Center, University of Pennsylvania, Philadelphia, USA

**Keywords:** adenocarcinoma in villous adenoma, national cancer database, ncdb, prognostic factors, socioeconomic factors

## Abstract

Purpose: Adenocarcinoma in villous adenoma is a rare malignancy arising from adenomatous polyps with a high villous content. This study aims to identify the demographic, prognostic, and socioeconomic factors influencing overall survival using data sourced from the National Cancer Database (NCDB).

Methods: Patients diagnosed with adenocarcinoma in villous adenoma were identified from the NCDB between 2004 and 2020. Kaplan-Meier curves, multivariable Cox proportional hazards models, life tables, and log-rank tests were used to assess the impact of variables such as age, sex, NCDB tumor stage, Charlson-Deyo (CD) comorbidity score, primary therapies, adjuvant therapies, and socioeconomic factors on survival.

Results: This cohort of 19,120 patients with adenocarcinoma in villous adenoma revealed multiple statistically significant factors affecting survival. Demographically, males had an 18% higher risk of mortality compared to females (hazard ratio (HR): 0.82; 95% confidence interval (CI): 0.79-0.86; p<0.001), older age (76-100 age group, five years: 45.3%; 10 years: 20.7%; p<0.001) correlated with poorer outcomes, and Black patients had a 22% increased mortality risk relative to White patients (HR: 1.22; 95% CI: 1.14-1.30; p<0.001). Socioeconomically, patients in lower-income brackets (<$46,277 vs. ≥$74,063, HR: 0.88; 95% CI: 0.82-0.94; p<0.001) and those with Medicare or Medicaid insurance faced increased mortality risks compared to those with private insurance (Medicare, HR: 1.26; 95% CI: 1.19-1.33; p<0.001; Medicaid, HR: 1.57; 95% CI: 1.41-1.74; p<0.001). Disease and comorbidity factors showed that higher CD comorbidity scores (10-year survival: 25.9% for CD 2; 17.8% for CD 3; p<0.001) and stage IV disease (HR: 9.23; 95% CI: 8.32-10.24; p<0.001) were associated with poorer outcomes. Regarding treatment, primary radiation increased mortality risk by 24% (HR: 1.24; 95% CI: 1.15-1.33; p<0.001), while neoadjuvant radiation and adjuvant chemotherapy decreased mortality risk by 39% (HR: 0.61; 95% CI: 0.54-0.69; p<0.001) and 26% (HR: 0.74; 95% CI: 0.68-0.81; p<0.001), respectively.

Conclusion: Adenocarcinoma in villous adenoma outcomes is influenced by age, sex, race, NCDB cancer stage, comorbidities, and treatment type. Surgical intervention remains the primary treatment and confers improved survival outcomes, emphasizing the importance of early detection and treatment. Further research, including prospective studies, is needed to validate these findings, investigate the mechanisms underlying observed disparities, and optimize treatment strategies, particularly the role of adjuvant therapies.

## Introduction

Adenocarcinoma in villous adenoma is a rare malignancy arising from premalignant polyps with ≥75% villous architecture [1-4]. The World Health Organization categorizes colorectal adenomas by villous content, that is, tubular (<25%), tubulovillous (25-75%), and villous (75-100%), with villous adenomas representing only 5% of cases [5]. These lesions are typically sessile, histologically characterized by elongated glandular projections exceeding twice the thickness of normal mucosa [5,6], and carry a high risk of malignant transformation (>50% when >2 cm). While most common in the rectosigmoid region, they may occur throughout the colon, underscoring the need for vigilant surveillance.

The clinical significance stems from their aggressive potential: up to 50% of large (defined as >2 cm) villous adenomas harbor malignancy [4,5]. Previous studies report five-year survival rates of 62-67% [7], with advanced age, tumor size, stage, and lack of surgical resection linked to poorer outcomes [5,7]. Surgery remains the cornerstone of treatment for larger or incompletely excised lesions [4], though optimal management strategies are poorly defined due to the condition's rarity. Notably, socioeconomic disparities and the impact of comorbidities on survival are understudied [8,9], limiting equitable care.

While adenocarcinoma arising from villous adenoma shares features with other colorectal cancers, its relative rarity and distinct histology necessitate specific investigation. Existing literature focuses primarily on histopathologic features and localized treatment outcomes [4,5,7], often lacking large-scale analyses that integrate diverse patient- and system-level factors. Critical gaps persist regarding the interplay of demographic characteristics, comorbidities, socioeconomic disparities (including insurance status, income, and education), and treatment modalities in determining survival for this specific malignancy. Using the National Cancer Database (NCDB), this study therefore undertakes a comprehensive investigation into the simultaneous influence of these varied factors on survival in adenocarcinoma arising from villous adenoma. By leveraging the largest cohort of adenocarcinoma arising from villous adenoma patients analyzed to date, this study aims not only to clarify predictors of mortality and confirm expected prognostic patterns but also to identify specific disparities in care access, outcomes, and any unique nuances pertinent to adenocarcinoma arising from villous adenoma. Findings may reinforce the importance of early detection, equitable resource allocation, and tailored therapeutic approaches, ultimately providing evidence directly applicable to this understudied patient population.

## Materials and methods

This is a retrospective cohort study of patients within the NCDB diagnosed with adenocarcinoma in villous adenoma from 2004 to 2020 [10]. The NCDB is a clinical oncology database derived from hospital registry data with information on patient characteristics, tumor staging, tumor histology, type of first treatment, disease recurrence, and survival from more than 1,500 Commission on Cancer (CoC)-accredited facilities. This de-identified patient data was made accessible to the authors through the Participant User Data Files program. 

Patients with adenocarcinoma in villous adenoma were identified from NCDB data using the International Classification of Diseases for Oncology, Third Edition (ICD-O-3), histology code 8261, which classifies tumors based on their morphological and histological features. Patients were then selected if they had behavior code 3 (invasive). 

Covariates

Patients were analyzed according to age, sex, race, education, income, insurance status, tumor size, analytical stage, Charlson-Deyo (CD) comorbidity score, primary anatomic site, surgery, adjuvant and primary radiation, adjuvant and primary chemotherapy, and distance traveled for healthcare. Race was categorized into three groups: White, African-American, and Other. Income was measured by median household income from 2016 to 2020 for the zip code where the patient resided at the time of diagnosis, as established by the American College of Surgeons (ACS). Education was measured in 2020 by the percentage of residents within the patient's zip code of residence who did not graduate from high school. Income and education were categorized into quartiles by the NCDB and ACS, and the specific cutoff values for each of the categories are shown in the Results section. The staging was measured by the NCDB analytical stage. Clinical staging was used when the variable NCDB analytical stage was not available. Insurance status was categorized into five groups: uninsured, private, Medicare, Medicaid, and other government insurance. The primary anatomical site was divided into seven groups using ICD-O-3 topography codes: cecum (C180), ascending colon (C182), transverse colon (C184), sigmoid colon (C187), rectosigmoid junction (C199), rectum (C209), and other. Distance traveled for healthcare was defined as the miles between the patient's residence and the hospital that reported the case. Treatment modalities were categorized based on NCDB standard procedures. Neoadjuvant radiation was identified using NCDB flags indicating radiation therapy completed prior to the primary surgical procedure. Adjuvant radiation was identified using NCDB flags indicating radiation therapy initiated following the primary surgical procedure. Adjuvant chemotherapy was defined similarly to adjuvant radiation, but for chemotherapy. Primary chemotherapy and radiation indicate that these therapies were the first-course treatments. The CD score, which measures comorbidities, was used to classify patients into groups with scores of 0, 1, 2, and ≥3. Tumor size and distance traveled were not included in the multivariable Cox proportional hazards regression and were therefore not quantized.

Overall survival, defined from the date of diagnosis until the date of death, was the primary outcome of interest. The data was censored by the date of last contact as reported by the NCDB. Kaplan-Meier curves were plotted, and overall survival was approximated at two-, five-, and 10-year intervals using survival tables. Multivariable Cox proportional hazards regression models were utilized to identify independent prognostic factors. The a priori variables included in the multivariable Cox model were age, sex, race, insurance status, education, income, CD comorbidity score, analytic stage, primary anatomic site, primary chemotherapy, primary radiation, adjuvant chemotherapy, and neoadjuvant radiation. Clustering of patients in the same facility was accounted for by employing a robust sandwich covariance matrix. The functional form of continuous variables was examined through locally estimated scatterplot smoothing (LOESS) methods, and the proportional hazards assumption for each variable was assessed using log-negative-log survival curves to test for statistical interaction with time. 

Statistical considerations

The data was evaluated using Cox proportional hazards regression models and Kaplan-Meier curves and life tables. Cox proportional hazards regression was chosen to analyze survival data because it effectively handles censored observations and assesses multiple prognostic factors at once. Kaplan-Meier curves were created to visually illustrate unadjusted survival differences, while life tables offered standardized survival estimates at key clinical time points (two, five, and 10 years). These methods align with the recommendations for cancer registry analysis provided by the NCDB and the American Joint Committee on Cancer (AJCC). 

The IBM SPSS Statistics for Windows, V. 27.0 (IBM Corp., Armonk, NY, USA), was used to analyze the descriptive statistics, unadjusted survival analysis, and multivariable analysis for this study. Patients with missing prognostic or demographic factors were excluded from the cohort. The Bonferroni correction was applied specifically to adjust the p-value threshold for the multiple pairwise comparisons conducted post hoc within categorical variables having more than two levels in the multivariable Cox model results presented in a table below (specifically for Race (three comparisons), CD score (three comparisons), Income (three comparisons vs. lowest), Insurance (four comparisons vs. Private), and Stage (three comparisons vs. Stage I)). This involved 16 comparisons, leading to an adjusted significance threshold of p<(0.05/16)=0.0031 to maintain control of the family-wise error rate at α=0.05 for these specific comparisons. Given the large sample size and the focus on establishing robust associations for these key predictors, this conservative approach was deemed appropriate for these pre-defined comparisons of interest, although we acknowledge that alternative methods like the false discovery rate (FDR) exist for broader exploratory contexts. Variance inflation factors (VIF) were calculated using linear regression models to assess multicollinearity among socioeconomic variables (income, education, and insurance status). All VIFs were below 2.0, under the threshold of 5.0, indicating there is no significant collinearity [8,11].

Oversight

The University of Arizona Biomedical Institutional Review Board (IRB) reviewed this study (IRB submission ID: STUDY00003534; approval number: 2001750-01) and determined that it does not involve human subjects research as defined by the Department of Health and Human Services (DHHS) and Food and Drug Administration (FDA) regulations. Consequently, IRB approval and ongoing review were not required.

## Results

The NCDB yielded an initial cohort of 30,960 patients diagnosed with adenocarcinoma in villous adenoma (ICD-O-3 code 8261) between 2004 and 2020. Initially, there were 5,797 (18.7%) patients with non-invasive (in situ) disease (behavior code ≠ 3), leaving 25,163 patients with invasive tumors potentially eligible for the study. An additional 6,043 patients (24% of the invasive cohort) were subsequently excluded due to missing information for one or more demographic, prognostic, or treatment variables required for the multivariable analysis. The final analytic cohort therefore comprised 19,120 patients with complete data. Descriptive statistics are displayed in Table 1. There was an approximately equal distribution of males and females, the majority of whom were White (85.1%; n=16,266). The cohort was predominantly composed of older adults, with a mean age of 67.7 years (95% CI: 67.48-67.86; standard deviation (SD): 13.123; standard error (SE): 0.095). A majority of patients presented with early-stage disease (51.5% at NCDB stage I; n=9,853) and low comorbidity burden (68.8% with a CD score of 0; n=13,157). In terms of socioeconomic status, 35.9% of the population fell into the highest-income bracket (≥$74,063; n=6,855), and nearly 21.6% of the population resided in zip codes with low educational attainment (≥15.3% without high school diplomas; n=4,124). Most patients relied on Medicare (56.1%; n=10,723), underscoring the population's age-related vulnerability. Patients were clustered geographically, with the largest proportions receiving treatment at facilities located in the South Atlantic (19%; n=3,558), East North Central area (17.4%; n=3,254), and Middle Atlantic (15.7%; n=2,938). The predominant facility type was comprehensive community cancer programs (45.7%; n=8,568) located in metropolitan areas with populations greater than or equal to one million or more individuals (53.3%; n=10,191).

**Table 1:** Clinical and demographic characteristics of 19,120 patients with adenocarcinoma in villous adenoma NCDB stage I: small, localized tumor. NCDB stage II: larger, localized tumor mass with slight lymph node and tissue involvement. NCDB stage III: large tumor with regional lymph node and tissue involvement. NCDB stage IV: metastatic cancer (cancer spread to distant parts of the body, e.g., brain, bone, liver, lungs, etc.) NCDB: National Cancer Database

Variable		N
Sex	Male	9554 (50%)
	Female	9566 (50%)
Race	White	16,266 (85.1%)
	Black	2068 (10.8%)
	Other	786 (4.1%)
Age (years)	Mean±standard deviation	67.67±13.12
	Median (interquartile range)	69.00 (20%)
Zip code-level median household income (2016-2020, $)	< $46,277	3395 (17.8%)
	$46,277-$57,856	4155 (21.7%)
	$57,857-$74,062	4715 (24.7%)
	≥$74,063	6855 (35.9%)
Zip code-level education (% without high school degree, 2020)	≥15.3%	4124 (21.6%)
	9.1-15.2%	5485 (28.7%)
	5-9%	5595 (29.5%)
	<5%	3916 (20.5%)
Insurance status	Uninsured	456 (2.4%)
	Private	6944 (36.3%)
	Medicaid	844 (4.4%)
	Medicare	10,723 (56.1%)
	Other government	153 (0.8%)
Distance traveled for healthcare (miles)	Mean±standard deviation	17.578±45.521
	Median (interquartile range)	7.8 (13.4%)
Charlson-Deyo comorbidity score	0	13,157 (68.8%)
	1	4064 (21.3%)
	2	1248 (6.5%)
	≥3	651 (3.4%)
Tumor size (mm)	Mean±standard deviation	42.064±43.296
	Median (interquartile range)	37 (35%)
NCDB analytical stage	I	9853 (51.5%)
	II	3608 (18.9%)
	III	3716 (19.4%)
	IV	1943 (10.2%)
Treatment	Received surgery	16,599 (86.8%)
	Received adjuvant chemotherapy	3441 (18%)
	Received neoadjuvant radiation	993 (5.2%)
Surgical margins	No residual tumor	16,361 (85.6%)	
	Residual tumor, not otherwise specified	287 (1.5%)	
	Microscopic residual tumor	436 (2.3%)	
	Macroscopic residual tumor	72 (0.4%)	

The primary sites of diagnosis were in the colon and/or rectum (23.9%; n=4,579), followed closely by the cecum (20.9%; n=4,000). The mean tumor size was 42 millimeters (SD: 43.296; SE: 1.119; 95% CI: 39.868-44.259). Surgery was the most common treatment modality, with 86.8% of patients undergoing tumor resection (n=16,599). The majority of patients had no evidence of residual tumor following surgery (85.6%; n=16,361). Postoperative survival rates were high, with 89.8% of patients surviving at 30 days (n=17,717) and 87.6% at 90 days (n=16,740). Other treatment modalities used on this cohort were primary chemotherapy (29.4%; n=5,627), adjuvant chemotherapy (18%; n=3,441), primary radiation (14.7%; n=2,803), and neoadjuvant radiation (5.2%, n=993). The majority of patients did not receive palliative care (98.3%; n=18,793).

**Figure 1:**
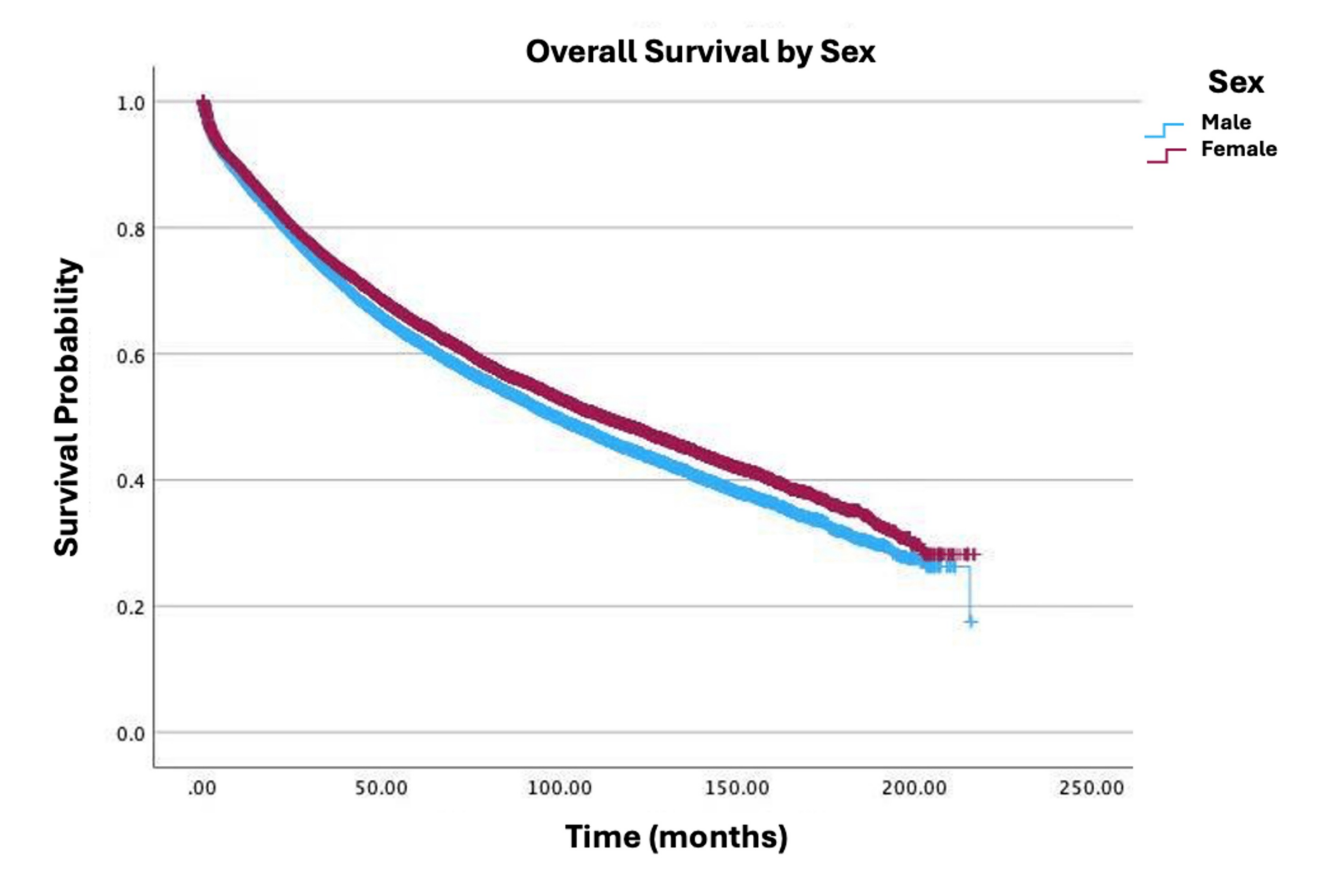
Overall survival by sex of patients with adenocarcinoma in villous adenoma (N=19,120; p<0.001)

**Figure 2:**
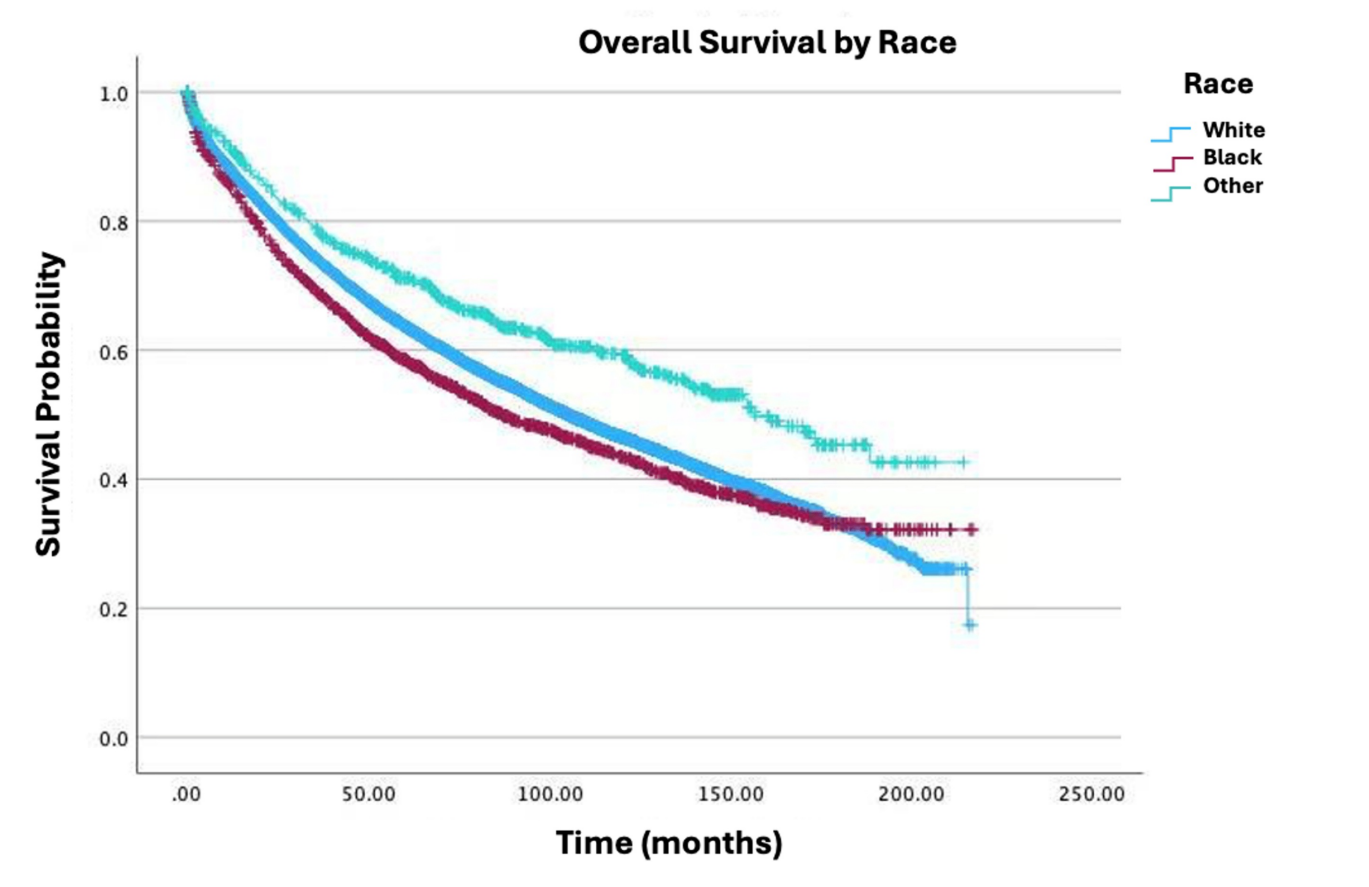
Overall survival by race of patients with adenocarcinoma in villous adenoma (N=19,120; p<0.001)

**Figure 3:**
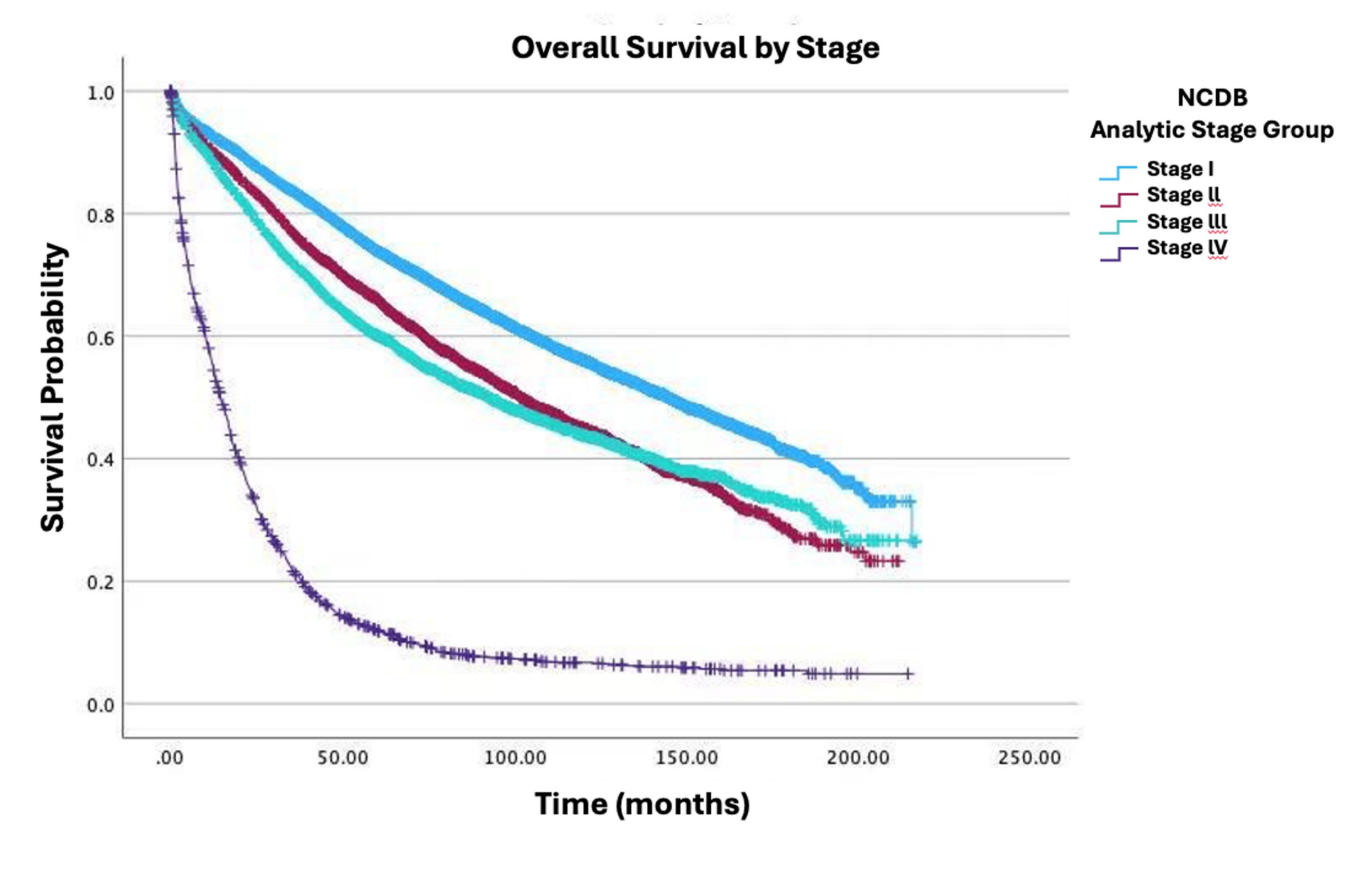
Overall survival by NCDB analytic stage of patients with adenocarcinoma in villous adenoma (N=19,120; p<0.001) NCDB stage I: small, localized tumor. NCDB stage II: larger, localized tumor mass with slight lymph node and tissue involvement. NCDB stage III: large tumor with regional lymph node and tissue involvement. NCDB stage IV: metastatic cancer (cancer spread to distant parts of the body, e.g., brain, bone, liver, lungs, etc.) NCDB: National Cancer Database

**Figure 4:**
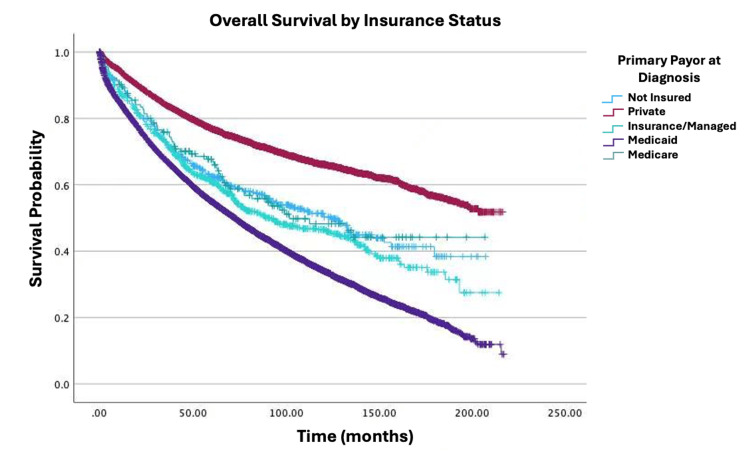
Overall survival by insurance status of patients with adenocarcinoma in villous adenoma (N=19,120; p<0.001)

Survival by specific variables is shown in Figures 1-4. Table 2 shows the two, five, and 10-year survival estimates by variable. Multivariable Cox model results are in Table 3. Upon multivariable Cox regression analysis, females had an 18% decreased risk of death when compared to males (HR: 0.82; 95% CI: 0.79-0.86; p<0.001). Older age was also associated with an increased hazard of death. Both five- and 10-year survival estimates declined with advancing age, with the lowest rates observed in the 76-100 age group (five years: 45.3%; 10 years: 20.7%; p<0.001). Higher CD comorbidity scores were correlated with reduced survival, as patients with a CD score of 2 had a 10-year survival rate of 25.9% versus 17.8% for those with a CD score of 3 (p<0.001). Stage IV disease conferred a 9.23-fold higher hazard of mortality compared to stage I (HR: 9.23; 95% CI: 8.32-10.24; p<0.001). Black patients had a 22% increased mortality risk relative to those of White descent (HR: 1.22; 95% CI: 1.14-1.30; p<0.001). Individuals in the lowest-income quartile (<$46,277) faced a 12% higher hazard of death when compared to the highest-income bracket ((≥$74,063) HR: 0.88; 95% CI: 0.82-0.94; p<0.001). Insurance status significantly influenced survival outcomes, with Medicare patients showing lower 10-year survival (34%) versus Medicaid (46.5%) and private insurance (66.1%). Medicaid and Medicare were associated with a 1.57-fold (HR: 1.57; 95% CI: 1.41-1.74; p<0.001) and 1.26-fold (HR: 1.26; 95% CI: 1.19-1.33; p<0.001) higher hazard of mortality, respectively, when compared to private insurance. Patients who received primary radiation had a 24% increased mortality risk (HR: 1.24; 95% CI: 1.15-1.33; p<0.001). Patients who received neoadjuvant radiation and adjuvant chemotherapy had a decreased mortality risk of 39% (HR: 0.61; 95% CI: 0.54-0.69; p<0.001) and 26% (HR: 0.74; 95% CI: 0.68-0.81; p<0.001), respectively. There were no any statistically significant relationships observed between high school education level and survival. 

**Table 2:** Median two-, five-, and 10-year survival estimates of 19,120 patients with adenocarcinoma in villous adenoma NCDB: National Cancer Database; N/A: not applicable

Variable		2-year N (%)	5-year N (%)	10-year N (%)
Sex	Male	7576 (79.3)	5933 (62.1)	4289 (44.9)
	Female	7739 (80.9)	6208 (64.9)	4639 (48.5)
Race	White	13078 (80.4)	10378 (63.8)	7564 (46.5)
	Black	1565 (75.7)	1210 (58.5)	900 (43.5)
	Other	N/A	N/A	N/A
Zip code-level median household income (2016-2020, $)	< $46,277	2614 (77)	1989 (58.6)	1423 (41.9)
	$46,277-$57,856	3278 (78.9)	2552 (61.4)	1841 (44.3)
	$57,857-$74,062	3810 (80.8)	3036 (64.4)	2240 (47.5)
	≥$74,063	5607 (81.8)	4559 (66.5)	3421 (49.9)
Zip code-level education (% without high school degree, 2020)	≥15.3%	3246 (78.7)	2532 (61.4)	1893 (45.9)
	9.1-15.2%	4361 (79.5)	3401 (62)	2463 (44.9)
	5-9%	4526 (80.9)	3631 (64.9)	2618 (46.8)
	<5%	3180 (81.2)	2577 (65.8)	1946 (49.7)
Age (years)	0-25	839 (87.8)	755 (79)	755 (79)
	26-50	2569 (89.6)	2272 (79.2)	2022 (70.5)
	51-75	6493 (84.9)	5408 (70.7)	4367 (57.1)
	76-100	5208 (68.1)	3464 (45.3)	1584 (20.7)
NCDB analytical stage	I	8671 (88)	7281 (73.9)	5528 (56.1)
	II	3020 (83.7)	2385 (66.1)	1624 (45)
	III	2958 (79.6)	2233 (60.1)	1628 (43.8)
	IV	653 (33.6)	231 (11.9)	130 (6.7)

**Table 3:** Multivariable Cox regression model of 19,120 patients with adenocarcinoma in villous adenoma NCDB: National Cancer Database; HR: hazard ratio

Variable		HR (95% CI)	P-values
Age (five years)		1.25 (1.24-1.27)	<0.001
Sex: males vs. females		0.82 (0.79-0.86)	<0.001
Race	White vs. Black	1.22 (1.14-1.30)	<0.001
	White vs. Other	0.75 (0.67-0.84)	<0.001
	Black vs. Other	0.62 (0.54-0.7)	<0.001
Charlson-Deyo comorbidity score	0 vs. 1	1.29 (1.23-1.36)	<0.001
	0 vs. 2	1.69 (1.57-1.81)	<0.001
	0 vs. ≥3	2.21 (2.01-2.42)	<0.001
Zip code-level median household income (2020, $)	< $46,277 vs. $46,227-$57,856	0.97 (0.91-1.03)	0.33
	< $46,277 vs. $57,857-$74,062	0.93 (0.87-0.99)	0.031
	< $46,277 vs. ≥$74,063	0.88 (0.82-0.94)	<0.001
Zip code-level education (2020, % no high school diploma)	≥15.3% vs. 9.1-15.2%	1.03 (0.97-1.09)	0.391
	≥15.3% vs. 5-9%	1.03 (0.96-1.10)	0.388
	≥15.3% vs. <5%	0.99 (0.92-1.06)	0.924
Insurance	None vs. private	0.62 (0.53-0.71)	<0.001
	None vs. Medicaid	0.96 (0.82-1.14)	0.678
	None vs. Medicare	0.78 (0.67-0.90)	<0.001
	None vs. other government	0.80 (0.61-1.06)	0.115
Treatment	No adjuvant chemotherapy vs. adjuvant chemotherapy	0.74 (0.68-0.81)	<0.001
	No adjuvant radiation vs. adjuvant radiation	0.61 (0.54-0.69)	<0.001
NCDB analytical stage	Stage I vs. stage II	1.35 (1.28-1.43)	<0.001
	Stage I vs. stage III	1.95 (1.83-2.08)	<0.001
	Stage I vs. stage IV	9.23 (8.59-9.90)	<0.001

## Discussion

This large-scale study utilizing the NCDB provides a thorough evaluation of demographic, clinical, socioeconomic, and treatment variables influencing overall survival in patients diagnosed with invasive adenocarcinoma arising in villous adenoma between 2004 and 2020. Our findings offer crucial insights into key determinants of outcomes for this relatively rare malignancy, providing much-needed large-scale data specific to this subtype. Consistent with broader patterns observed in some gastrointestinal cancers [12], our cohort showed a nearly even distribution between males and females. However, males exhibited significantly poorer survival outcomes compared to females (HR: 0.82 for females vs. males) in our adjusted analysis, a finding observed in some other cancers that warrants further investigation into potential biological or behavioral factors. The rectum and cecum were the predominant primary tumor sites, aligning with known distributions of colorectal adenocarcinoma [13]. The anatomical location within the colorectum can influence treatment approaches and prognosis, with rectal cancers often presenting unique challenges for surgical resection and radiotherapy [14].

As expected, stage at diagnosis was identified as a critical factor for survival, with the majority of patients diagnosed at stage I, emphasizing the importance of early detection. This finding is consistent with prior research linking early-stage diagnosis to improved survival rates [15]. Advanced-stage disease was associated with a 9.23-fold increase in mortality risk, which corroborates existing evidence highlighting the drastic survival decline associated with metastatic disease [16]. Tumor size also emerged as a prognostic factor, with larger tumors linked to poorer outcomes. The average tumor size in this cohort was 42 millimeters, consistent with studies showing that smaller tumors, detected earlier, often lead to better survival outcomes and highlight the value of effective screening initiatives [17].

Treatment patterns observed in the study underscore the value of multimodal approaches. Surgery was the primary intervention, performed in nearly 90% of treated patients, reaffirming its central role in managing invasive tumors [18]. Both 30- and 90-day postoperative survival rates were high, underscoring the impact of surgical expertise and advancements in perioperative care [19]. The interpretation of findings related to non-surgical treatments requires the careful consideration of potential confounding by indication. Our analysis showed that receiving primary radiation was associated with a 24% increased mortality risk (HR: 1.24). This counterintuitive result is highly likely attributable to patient selection bias; this group may disproportionately include individuals ineligible for surgery due to advanced unresectable disease or significant comorbidities or those receiving radiation with palliative rather than curative intent [20]. In stark contrast, neoadjuvant radiation (HR: 0.61) and adjuvant chemotherapy (HR: 0.74) were associated with significantly decreased mortality risk. This likely reflects their established roles within curative-intent, multimodality treatment regimens for appropriately selected patients with locally advanced (neoadjuvant radiation, typically rectal/rectosigmoid) or higher-risk resected disease (adjuvant chemotherapy) [21]. These therapies are generally offered to patients deemed fit enough to tolerate them and expected to derive benefit; thus, the observed survival advantage incorporates both treatment effect and patient selection factors. Determining the precise causal impact of these adjuvant/neoadjuvant therapies would necessitate study designs that rigorously control for selection bias, such as propensity score matching or randomized trials, though the latter is challenging for rare tumor subtypes.

Our study also revealed significant socioeconomic and racial disparities influencing survival. Black patients experienced a 22% higher mortality risk compared to White patients, even after adjusting for other factors, including stage and comorbidities. This finding adds to the body of evidence demonstrating persistent racial disparities in cancer outcomes, potentially stemming from a complex interplay involving differential access to high-quality care, systemic biases, socioeconomic pressures, mistrust, transportation barriers, higher prevalence of unmeasured comorbidities, and potentially underlying biological differences, demanding further investigation [8,9,22,23]. Furthermore, socioeconomic status displayed intricate associations with outcomes, consistent with prior research highlighting its multifaceted influence in cancer care [24]. Patients residing in lower-income zip codes (<46,277) faced worse survival compared to those in the highest-income bracket (≥74,063). This disparity likely reflects multifaceted barriers associated with lower income, potentially including reduced access to specialist care, difficulties adhering to complex treatment regimens due to cost or logistical challenges, food insecurity, exposure to environmental stressors, and delayed diagnosis, all of which can negatively impact survival outcomes [24,25]. Insurance type was also a powerful predictor, with patients covered by Medicare or Medicaid experiencing significantly higher mortality risk compared to those with private insurance. This underscores systemic issues related to underinsurance and potential disparities in the quality, intensity, or timeliness of care received across different insurance types, even after accounting for age and comorbidities. Interestingly, zip code-level educational attainment (% without high school diploma) was not significantly associated with survival in our multivariable model, which contrasts with some studies linking individual-level education to health literacy and outcomes [26]. This discrepancy might reflect the limitations of using an area-based measure for education, the stronger influence of income and insurance in this specific cohort, or potentially that education's impact is mediated through income and insurance, which were included in the model. Collectively, these findings highlight the critical need for targeted interventions and policies aimed at ensuring equitable access to timely diagnosis, high-quality treatment, and supportive care for all patients.

Limitations

This study has several inherent limitations common to retrospective database analyses using the NCDB. Our reliance on the NCDB restricts the analysis to pre-defined variables, potentially omitting granular clinical details (e.g., specific chemotherapy regimens, performance status metrics beyond the CD score, detailed surgical margin notes, specific reasons for treatment choices, or molecular tumor characteristics) that could influence outcomes. Consequently, despite multivariable adjustment, residual confounding from unmeasured factors remains a possibility, and the retrospective design precludes the establishment of definitive causal relationships.

Furthermore, our analysis excluded patients with missing data for key covariates used in the multivariable model (n=6,043, representing 24% of the potentially eligible invasive cohort). This complete case analysis could potentially introduce selection bias if the characteristics of patients with missing data systematically differ from those with complete data.

A significant limitation is the NCDB's reporting of overall survival rather than cancer-specific survival (CSS). This prevents the differentiation between mortality directly attributable to adenocarcinoma in villous adenoma and deaths resulting from other causes. This is particularly relevant given the cohort's mean age and the prevalence of comorbidities, where non-cancer-related mortality can be substantial. The inability to isolate cancer-specific death may dilute the observed effects of tumor-related prognostic factors and complicates the interpretation of associations with factors like comorbidities or socioeconomic status, where competing risks of death are significant.

Interpreting the associations with treatment modalities requires particular caution due to the strong potential for confounding by indication, which statistical adjustment may not fully eliminate. This is particularly evident in the divergent findings for primary versus neoadjuvant radiation therapy discussed earlier. The observed associations likely reflect fundamental differences in patient populations, disease characteristics, and treatment goals between the groups receiving different treatments, rather than providing a direct comparison of efficacy in equivalent patients.

The restriction of NCDB data to CoC-accredited facilities introduces potential selection bias and may limit the generalizability of our findings to patients treated in non-accredited institutions. Although CoC accreditation has been associated with adherence to quality metrics and potentially improved survival for certain cancers [24-26], the extent to which outcomes may differ in non-CoC settings for this specific malignancy is unknown.

Finally, the use of zip code-level data for median household income and education level introduces the potential for ecological fallacy, as these area-based measures may not accurately represent the socioeconomic status of individual patients within those zip codes.

Despite these limitations, this study utilizes a large, contemporary, nationwide cohort, providing valuable insights into the factors influencing survival in this rare malignancy within the context of the US healthcare system represented by CoC-accredited centers.

## Conclusions

This study highlights key prognostic factors for adenocarcinoma in villous adenoma, including advanced age, higher comorbidity scores, larger tumor size, and advanced-stage disease, which were all associated with poorer survival outcomes. While confirming the importance of factors known in broader colorectal cancer cohorts, this research provides robust, specific quantification of these effects within the context of this rare malignancy. Socioeconomic disparities significantly influenced survival, with private insurance and higher income levels correlating with better outcomes. Surgical resection remains the primary curative modality. While potential confounding by indication must be considered, particularly for primary radiation (associated with increased mortality risk), neoadjuvant radiation and adjuvant chemotherapy were associated with significantly improved survival outcomes in this analysis, highlighting their potential roles in multimodality treatment for selected patients. These findings underscore the importance of early detection, equitable healthcare access, and tailored interventions to improve outcomes for adenocarcinoma in villous adenoma. Further research is warranted to refine treatment approaches and address disparities in care for this large, specific patient group.
